# Prognostic Value of ^18^F-Fluorocholine PET Parameters in Metastatic Castrate-Resistant Prostate Cancer Patients Treated with Docetaxel

**DOI:** 10.1155/2019/4325946

**Published:** 2019-03-26

**Authors:** E. Quaquarini, D. D'Ambrosio, F. Sottotetti, F. Gallivanone, M. Hodolic, P. Baiardi, R. Palumbo, C. Vellani, C. Canevari, A. Bernardo, I. Castiglioni, C. Porta, G. Trifirò

**Affiliations:** ^1^Medical Oncology Unit, ICS Maugeri SpA SB-IRCCS, Pavia 27100, Italy; ^2^University of Pavia, Ph.D. in Experimental Medicine, Pavia 27100, Italy; ^3^Medical Physics Unit, ICS Maugeri SpA SB-IRCCS, Pavia 27100, Italy; ^4^Institute of Molecular Bioimaging and Physiology, National Research Council (IBFM-CNR), Milan 20090, Italy; ^5^Nuclear Medicine Research Department, Iason, Graz, Austria; ^6^Nuclear Medicine Department, Faculty of Medicine and Dentistry, Palacký University Olomouc, Olomouc, Czech Republic; ^7^Scientific Direction, ICS Maugeri SpA SB-IRCCS, Pavia 27100, Italy; ^8^Nuclear Medicine Unit, ICS Maugeri SpA SB-IRCCS, Pavia 27100, Italy; ^9^Nuclear Medicine Unit, IRCCS San Raffaele Scientific Institute, Milan 20132, Italy; ^10^Translational Oncology Unit, ICS Maugeri SpA SB-IRCCS, Pavia 27100, Italy; ^11^University of Pavia, Department of Internal Medicine, Pavia 27100, Italy

## Abstract

**Background and Aim:**

The availability of new treatments for metastatic castrate-resistant prostate cancer (mCRPC) patients increases the need for reliable biomarkers to help clinicians to choose the better sequence strategy. The aim of the present retrospective and observational work is to investigate the prognostic value of ^18^F-fluorocholine (^18^F-FCH) positron emission tomography (PET) parameters in mCRPC.

**Materials and Methods:**

Between March 2013 and August 2016, 29 patients with mCRPC were included. They all received three-weekly docetaxel after androgen deprivation therapy, and they underwent ^18^F-FCH PET/computed tomography (CT) before and after the therapy. Semi-quantitative indices such as maximum standardized uptake value (SUV_max_), mean standardized uptake value (SUV_mean_) with partial volume effect (PVC-SUV) correction, metabolically active tumour volume (MATV), and total lesion activity (TLA) with partial volume effect (PVC-TLA) correction were measured both in pre-treatment and post-treatment ^18^F-FCH PET/CT scans for each lesion. Whole-body indices were calculated as sum of values measured for each lesion (SSUV_max_, SPVC-SUV, SMATV, and STLA). Progression-free survival (PFS) and overall survival (OS) were considered as clinical endpoints. Univariate and multivariate hazard ratios for whole-body ^18^F-FCH PET indices were performed, and *p* < 0.05 was considered as significant.

**Results:**

Cox regression analysis showed a statistically significant correlation between PFS, SMATV, and STLA. No correlations between OS and ^18^F-FCH PET parameters were defined probably due to the small sample size.

**Conclusions:**

Semi-quantitative indices such as SMATV and STLA at baseline have a prognostic role in patients treated with docetaxel for mCRPC, suggesting a potential role of ^18^F-FCH PET/CT imaging in clinical decision-making.

## 1. Introduction

Prostate cancer (PC) is the first most common cancer in men worldwide, and its incidence is increasing in countries of higher socioeconomic development [1, 2].

The condition of metastatic castrate-resistant prostate cancer (mCRPC) has a poor outcome with a median overall survival (OS) of 20 months; thus, beyond palliation of symptoms and maintenance of a good quality of life, prolongation of survival remains largely an elusive goal [3, 4]. During the last decades and particularly in the past few years, chemotherapeutic agents (docetaxel and cabazitaxel) [5], antiandrogen drugs (abiraterone and enzalutamide) [6, 7], and radium-223 dichloride have been introduced for the treatment of mCRPC [8]. The optimal timing of the different treatments has not yet been established, due to the paucity of prognostic markers for sequence and clinical management decisions.

During treatment of mCRPC, the use of PSA, a glycoprotein mainly produced by prostate tissue, as a marker of response should be carefully evaluated. PSA fluctuations are well known and described during active treatments, often not due to a disease progression but to the effects of drugs on PSA production. In addition, conventional radiology has limitation in detecting tumour biology and behaviour during treatment. Positron emission tomography/computed tomography (PET/CT) has been widely used for the evaluation of PC by using several radiopharmaceuticals. A recent paper by Wallit and colleagues analyses the clinically available PET radiotracers for PC imaging and their mechanisms of actions: ^18^F-fluorodeoxyglucose (^18^F-FDG) for the evaluation of treatment response in metastatic bone disease; ^18^F-sodium fluoride (^18^F-NaF) for bone metastasis detection; ^11^C-choline and ^18^F-choline (^18^F-FCH) for the staging of high-risk patients and in presence of biochemical relapse with high PSA levels; ^68^Ga-labeled prostate-specific membrane antigen (^68^Ga-PSMA) highly sensitive for biochemical relapse with low PSA levels; the novel ^18^F-fluciclovine (^18^F-FACBC) appearing superior to choline in the setting of biochemical relapse [9–11].

There are several data in literature regarding the use of ^11^C-choline PET/CT in patients with PC treated with docetaxel both in neadjuvant and advance setting [12, 13], but its role for the assessment of the treatment response and for predicting patient outcome still remains unclear.

In comparison to CT, ^18^F-FDG PET/CT, and ^11^C-choline PET/CT, ^18^F-FCH PET/CT showed higher sensitivity and specificity in detection of metastatic lesions in patients with PC [14]. Its role in treatment monitoring and outcome prediction beyond PSA response of patients with mCRPC treated with abiraterone and enzalutamide has been already described [15–18].

To our knowledge, there are no data available on the use of ^18^F-FCH PET parameters as prognostic markers in patients affected by PC and treated with docetaxel for advanced disease.

The aim of this study was to explore the prognostic role of ^18^F-FCH PET/CT in patients treated with three-weekly docetaxel for mCRPC by using accurate PET biomarkers corrected for partial volume effect (PVE) [19].

## 2. Materials and Methods

### 2.1. Patients and Study Design

The present study is a retrospective, monocentric, observational trial that involved consecutive patients with a histological diagnosis of prostate cancer fulfilling PCWG3 criteria [20] for CRPC (baseline serum testosterone <50 ng/dl, progressive disease to androgen deprivation therapy).

This study was approved by the institutional ethics committee, and all patients signed written informed consent. The research was conducted according to the principles of the Declaration of Helsinki.

We considered eligible patients who had not yet received chemotherapy for advance disease, had a measurable disease according to PET Response Criteria in Solid Tumours (PERCIST) version 1.0 [21], and had an Eastern Cooperative Oncology Group (ECOG) performance status (PS) ≤ 2 and appropriate cardiac, hepatic, renal, and bone marrow function.

All patients underwent ^18^F-FCH PET/CT before (PET1) and after (PET2) the programmed chemotherapy treatment. The study was conducted between March 2013 and August 2016. Data collection ended on 31st of December 2017 for analysis. The primary objective of this study is the identification of ^18^F-FCH PET parameters that can predict clinical outcome in patients with mCRPC. As secondary objective, we evaluated the correlations between patients' clinical parameters and outcome.

#### 2.1.1. Chemotherapy Protocol

Docetaxel was administered in three-weekly schedule (75 mg/m^2^ day 1 every 21 days) as standard first-line chemotherapy for mCRCP according to the current guidelines [22]. The treatment was administered for a total of six cycles, and it was infused if clinical and biochemical parameters were permissive (conserved PS and no grade 3 or 4 adverse events according to Common Terminology Criteria for Adverse Events v4.03). Before the beginning of the therapy, patients underwent a baseline blood PSA assessment; PSA response and toxicity were evaluated before every dose administration. Dose adjustments and delays were planned to correspond with the type and grade of the observed toxicity. Concomitant medications such as antiemetic drug, granulocyte colony stimulating factors (G-CSF) for the secondary prevention of neutropenic fever, bisphosphonate treatments, and steroids were allowed.

#### 2.1.2. ^18^F-FCH PET/CT Procedure

PET/CT was performed after intravenous injection of ^18^F-FCH (IASOcholine®, Graz, Austria), according to the body weight of the patient (about 3.5 MBq/kg). The mean radiopharmaceutical dose injected to the patients was 213 MBq (range: 176–346 MBq). ^18^F-FCH PET/CT images were acquired in median 22 days (range: 8–38) before starting the treatment with docetaxel and in median 26 days (range: 14–58) after the treatment.

Images' acquisition was performed after an uptake time of approximately 60 min on a Discovery-690 VCT (General Electric Medical Systems, GEMS, Milwaukee, WI) scanner [23].

^18^F-FCH PET/CT data were acquired for 3 minutes per bed position and reconstructed by using OSEM algorithm (3 iterations, 18 subsets, full width at half maximum (FWHM) of smoothing filter equals to 5 mm) including time of flight and resolution recovery.

### 2.2. ^18^F-FCH PET/CT Image Analysis and Interpretation

A qualitative evaluation of ^18^F-FCH PET/CT images was firstly performed by two nuclear medicine physicians, and then an expert nuclear medicine physician selected the lesions easily identifiable and accurately evaluable on ^18^F-FCH PET/CT images before and after the chemotherapy in order to be semi-quantified. Too small lesions (volume < 1 cm) and lymph node clusters were excluded.

Semi-quantitative analysis of PET/CT lesions was performed by using a validated segmentation method [24]. ^18^F-FCH PET parameters were extracted considering partial volume effect correction (PVC) in order to get accurate PET biomarkers by using a validated method developed by Gallivanone and colleagues [25, 26]. More precisely, the following parameters were extracted from each segmented lesion on pre-treatment and post-treatment ^18^F-FCH PET images: maximum standardized uptake value (SUV_max_), mean standardized uptake value (SUV_mean_) corrected for PVE (PVC-SUV), metabolically active tumour volume (MATV), and total lesion activity (TLA). TLA was calculated as MATV multiplied by the PVC-SUV (TLA = MATV × PVC-SUV), and thus, TLA was corrected for PVE.

In order to extract whole-body indices for each patient and each ^18^F-FCH PET parameter (SUV_max_, PVC-SUV, MATV, and TLA), the sum of values measured for each lesion was calculated (SSUV_max_, SPVC-SUV, SMATV, and STLA, respectively).

### 2.3. Statistical Analysis

The distributions of the categorical variables are described by counts and frequencies or by median and range, whereas those of continuous and count variables are described by median and interquartile range. PFS was defined as the time between the date of the beginning of docetaxel and the date of progression. OS was defined as the time between the date of the starting of docetaxel and the date of death or last follow-up.

Univariate and multivariate hazard ratios for selected potential predictors of PFS and OS were performed using a Cox proportional hazards regression model. PFS and OS were estimated using the Kaplan–Meier method. *p* < 0.05 was considered as significant for all analyses. All statistical analyses were performed using SPSS version 24.

## 3. Results

### 3.1. Patients and Study Design

We enrolled 29 patients, whose baseline characteristics are reported in Table 1. Patients' median age at the beginning of docetaxel treatment was 71 (range: 42–82). In a median follow-up period of six years, 14% (*n*=4) patients obtained a complete metabolic response (CMR); 52% (*n*=15) a partial metabolic response; 7% (*n*=2) a stable metabolic disease; 27% (*n*=8) a progressive metabolic disease (PMD) as best response to treatment. Median PFS was 13.5 months (range 2.3–37.6 months), and median OS was 37 months (range 4.7–66 months). A PSA increase compared to baseline was seen in 5 patients (17%), whereas a PSA decline ≥50% was seen in 14 patients (47%). At the time of the analysis, 15 patients were still alive.

### 3.2. ^18^F-FCH PET/CT Image Analysis and Interpretation

**Figure 1:**
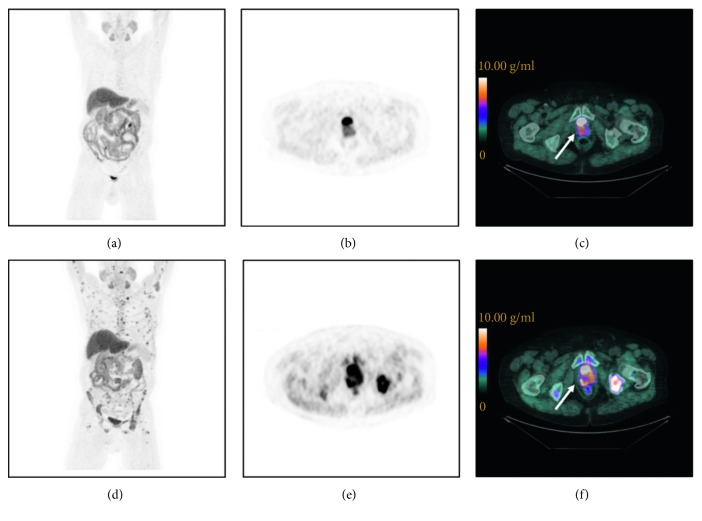
^18^F-FCH PET/CT images of a mCRPC patient (83 years old) with a PMD after docetaxel treatment. Baseline (a–c) and post-treatment (d–f) images. Maximum intensity projection images (a, d), transaxial slice of PET images (b, e), and PET/CT images (c, f) showing the prostate (arrows): pre-treatment SUV_max_ = 8.8 g/ml (c) and post-treatment SUV_max_ = 12.0 g/ml (f).

**Figure 2:**
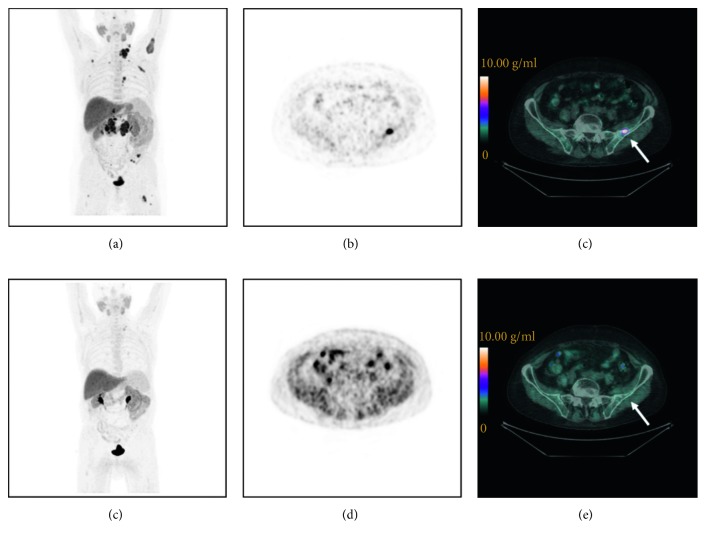
^18^F-FCH PET/CT images of a mCRPC patient (73 years old) who underwent prostatectomy and obtained a CMR to docetaxel treatment. Baseline (a– c) and post-treatment (d–f) images. Maximum intensity projection images (a, d), transaxial slice of PET images (b, e), and PET/CT images (c, f) showing a metastasis in the left iliac bone (arrows) before docetaxel treatment: SUV_max_ = 14.6 g/ml (c); after treatment, metastasis disappeared.

Seventy-one metastatic lesions were analysed, and semi-quantitative parameters were measured. Whole-body indices calculated for each patient before and after treatment are summarized in Table 2. Overall, 10% of patients had an SPVC-SUV reduction <25%, 24% had 25–74% reduction, and 10.3% had ≥75% reduction; 14% had an SSUVmax reduction <25%, 34% had 25–74% reduction, and 17% had ≥75% reduction; 17% had an SMATV reduction <25%, 38% had 25–74% reduction, and 17% had ≥75% reduction; and 21% had an STLA reduction <25%, 35% had 25–74% reduction, and 17% had ≥75% reduction. Figures 1 and 2 represent an example of PET/CT images showing a typical ^18^F-FCH distribution in a patient with PMD and CMR, respectively.

### 3.3. Statistical Analysis

As concerning the primary objective, at Cox regression analysis, SMATV and STLA ^18^F-FCH PET parameters were significantly correlated with PFS (HR = 1.069, 95% CI: 1.06–1.09, *p*=0.005 and HR 1.04, 95% CI: 1.02–1.08, *p*=0.012, respectively). No statistical significant correlations were found with OS (Table 3).

Since there was a statistically significant association between SMATV and PFS, a ROC curve analysis was performed, and it showed that patients with an SMATV value >27 cc at PET1 have a 20% higher probability of having progression during docetaxel treatment (HR 1.19, range 0.56–2.53, *p*=0.63) with a sensibility value of 73%, a specificity value of 58%, and an AUC of 0.64 (*p*=0.23).

As regarding the secondary objectives, a significant correlation between PSA and PFS was found since patients with a PSA decline ≥50% had a better outcome (median PFS 12.8 months, range 9.5–15.8) than patients with a PSA decline <50% (median PFS 9.7 months, range 9.5–13.7) (log rank test = *p* < 0.001). Patients with a PSA increase of during docetaxel treatment had a poor outcome (median PFS 6.2 months, range 5.1–7.2, *p*=0.036).

PSA decline was also correlated with OS since patients with a PSA decline ≥50% had a median OS of 42 months (range 34–49.53), patients with a PSA decline <50% had a median OS of 29.6 months (range 26.7–32.4), and patients with a PSA increase of had a reduced OS (median OS 26.3 months, range 25.8–26.7). The overall log-rank test, however, resulted not significant (*p*=0.56) maybe due to the small number of analysed events.

Patients with an age ≥65 years at the time of the starting of docetaxel treatment had a better outcome than younger ones (PFS 10.9 months, range 7.7–14.2, vs 6.6 months, range 4.8–8.3, respectively, *p*=0.054; OS 42 months, range 33.2–50.7, vs 28.5 months, range 22.5–34.4, respectively, *p*=0.076).

Considering the metastatic sites, patients with bone and lymph node lesions had a better outcome than patients with visceral involvement (PFS 11.6 months, range 9.6–13.3, vs 9.1 months, range 5.2–12.9, respectively, *p*=0.04; OS 38.4 months, range 34.8–41.9, vs 29.4 months, range 5.2–53.9, respectively, *p*=0.25).

## 4. Discussion

To our knowledge, this is the first study that evaluated the role of ^18^F-FCH PET uptake before and after docetaxel treatment as a means of predicting long-term clinical outcomes in mCRPC.

Docetaxel is one of the treatment options in patients affected by metastatic PC; however, no approved biomarkers can predict the outcome to this therapy. ^11^C-Choline and ^18^F-FCH PET/CT are widely used diagnostic techniques, and recent studies have evaluated the role of ^18^F-FCH PET indices in predicting treatment outcomes in CRPC patients [15–18, 27]. For the first time, Kwee and colleagues [28] assessed the potential usefulness of ^18^F-FCH PET parameters in mCRPC patients, quantifying whole-body tumour burden on the basis of SUV_max_, metabolic tumour volume (MTV), and TLA. They found that MTV and TLA measurements proved to strongly correlate in the Kaplan–Meyer analysis.

Afterwards, Caroli and colleagues retrospectively evaluated ^18^F-FCH PET parameters in 94 patients treated with enzalutamide or abiraterone for mCRPC [16]. At univariate analysis, they described that the median sum of MTV (SMTV), SUV_max_ (SSUV_max_), and TLA (STLA) resulted significant for OS and PFS, whereas, in multivariate analysis, only STLA remained statistically significant with an HR = 1.49 for PFS (95% CI 1.24–1.78, *p* < 0.001) and an HR = 1.46 for OS (95% CI 1.16–1.84, *p* = 0.001).

Another study by Maines and colleagues enrolled 30 patients treated with enzalutamide and demonstrated, in multivariate analysis, a statistically significant correlation between baseline mean SUV_max_ and PFS and between baseline mean SUV_max_ and OS: patients with higher baseline mean SUV_max_ experienced reduced PFS and OS than those with lower values (median PFS: 4 vs 8 months; HR: 1.22; 95% CI: 1.09–1.37; *p* < 0.0001; median OS: 12 months vs not reached, HR: 1.21; 95% CI: 1.01–1.44; *p*=0.03) [17].

De Giorgi and colleagues evaluated the utility of ^18^F-FCH PET parameters to detect an early response to abiraterone [15]. The authors concluded that a radiologic response to ^18^F-FCH PET/CT was associated to a better outcome compared to having obtained only a biochemical response.

Recently, Ceci and colleagues [29] have assessed the role of ^11^C-choline PET/CT to determine the response to docetaxel in a cohort of 61 patients with metastatic PC. The authors compared the radiologic response obtained with ^11^C-choline PET/CT and PSA response. The study had demonstrated incongruent results between the two methodologies since a radiologic progression was observed in 44% of patients with a biochemical response.

Another study by Schwarzenböck and collegues [13] evaluated the relationship between changes of SUV_max_ and SUV_mean_ of ^11^C-choline PET as a predictive biomarker of early and late response to docetaxel treatment in mCRCP. However, they did not find any significant correlation between the changes in choline uptake and the objective responses evaluated with RECIST and clinical criteria.

The results from our study suggest that ^18^F-FCH PET parameters could be used to predict the clinical outcome of patients with mCRPC treated with docetaxel after progressing to androgen-deprivation therapy. In particular, SMATV and STLA ^18^F-FCH PET parameters are the most promising imaging biomarkers, taking into account the metabolic tumour volume and activity. In fact, their mean baseline values seem to predict long-term clinical outcomes, thus suggesting that metabolic imaging may be useful to select the best treatment for individual patients and open new perspectives in clinical decision-making. Imaging biomarkers may help to tailor treatments as patients with higher levels of basal metabolic activity (and therefore a poorer prognosis) may benefit from more aggressive treatments. In this context, it is still uncertain whether pre-treatment metabolic imaging may also play a predictive role.

Our study benefits also from the use of a validated method to obtain tumour metabolic volume. As underlined in different publications and in particular in a work by Soret and colleagues [19], proper tumour active metabolic region assessment is paramount because it influences the measurement of different semi-quantitative indices to be evaluated as imaging biomarkers. In this study, we used a segmentation procedure that was validated on lesions that reliably simulate realistic tumour conditions (non-spherical and non-homogeneous), estimating volume with 92% of accuracy [24]. Furthermore, in order to obtain accurate quantitative indices of glucose consumption, a PVC method was applied to quantitative indices ensuring an accuracy for quantification up to 93% for lesions >1 cm as sphere-equivalent diameter.

We did not find any correlation between ^18^F-FCH PET parameters and survival. This is possibly due to the small sample size and the few number of events at the time of data analysis; as already well known from the literature, our findings demonstrated the prognostic role of visceral metastasis involvement.

## 5. Conclusions

Our findings suggest that ^18^F-FCH PET parameters, such as SMATV and STLA at baseline, have a prognostic role in patients treated with docetaxel for mCRPC and may be more useful than commonly used PET indices such as SUV_mean_ and SUV_max_. The study has some limitations due to the retrospective nature, the small sample size, and the single institution setting. Further investigation and larger studies are needed in order to find a significant correlation between ^18^F-FCH PET indices and OS.

## Figures and Tables

**Table 1:** Demographic and clinical characteristics of patients with CRPC at baseline (*n*=29).

	Median (range) or no.	%
Age	71 (42–82)	

ECOG		
0	18	62
1	9	31
2	2	7

Gleason score		
6-7	10	34
8-9	17	59
10	2	7

Baseline PSA		
Median (range) (ng/ML)	54.4 (0.93–361)	

Type of metastatic sites		
Lung	2	
Liver	3	
Only bone	24	
Only lymph nodes	23	
Bone and lymph nodes	8	
Local	5	

No. of previous treatment for CRPC disease		
None	5	17
One	21	73
Two or more	3	10

**Table 2:** Whole-body semi-quantitative ^18^F-FCH PET parameters before (PET1) and after (PET2) treatment (median (IQR)).

	PET1	PET2
SPVC-SUV (g/cc)	21.3 (10.8–31.2)	9.1 (4.2–26.5)
SSUV_max_ (g/cc)	26.2 (11.2–29.9)	8.8 (3.8–22.7)
SMATV (cc)	27.1 (12.1–48.0)	14.2 (1.4–32.3)
STLA (g)	253.5 (94.7–469.8)	118.9 (12.3–251.4)

**Table 3:** Cox regression analysis for progression-free survival and overall survival according to PET parameters.

Cox regression analysis						
Parameters	PFS			OS		
	HR	95% CI	*p*	HR	95% CI	*p*
SPVC-SUV ^18^F-FCH PET/CT	1.025	0.99–1.05	0.12	1.07	0.96–1.05	0.76
SSUV_max_^18^F-FCH PET/CT	1.022	0.99–1.05	0.118	1.03	0.97–1.03	0.85
SMATV ^18^F-FCH PET/CT	1.069	1.06–1.09	0.005	1.01	0.99–1.02	0.09
STLA ^18^F-FCH PET/CT	1.04	1.02–1.08	0.012	1.0	0.99–1.01	0.38

## Data Availability

The ^18^F-FCH PET/TC data used to support the findings of this study are available from the corresponding author upon request.
